# Medical Charities

**Published:** 1880-03

**Authors:** 


### Editorial.

### MEDICAL CHARITIES.

The abuse of medical charities has been the subject of discussion in nearly all the larger cities, both in this country and in England. Recently it has been attracting more attention than usual in this city, and is at present the subject of frequent discussion, especially among the younger members of the profession. The general social problem of how best to aid the really needy without encouraging idleness, pauperism and vice, is one of great importance and its practical solution involved in many difficulties. The medical aspect of the problem, or that which relates to the professional care of the sick poor, is the one that more immediately interests our readers, and indeed the profession generally. That the establishment of free hospitals, dispensaries, infirmaries, institutes, college clinics, etc., and liberally supplying them with medical and surgical attendants and medicines, tends strongly to increase the number of applicants for medical charity, and in the same proportion to increase the burden of taxation, on the one hand, and on the other to rob the members of the medical profession of the patronage of a large number of patients who are really able to pay moderate fees for all the attendance they need, has been so fully demonstrated in all the larger cities of our country as to need no further discussion.

When more than one out of every twenty of the entire population of a city like Chicago, is found to be receiving gratuitous medical services in connection with the various free dispensaries, it needs no further argument to show that a great wrong is being inflicted, both on the taxpayers and the members of the medical profession, while the undeserving recipients of the charity are themselves injured in the impairment of their feeling of self-reliance, independence and motives to industry. Many in the profession are disposed to attribute all the evils and abuses to which we allude to the lax and careless manner in which the public medical charities are administered, and especially to the ambition of those connected with these charities and with the medical colleges, to draw to them as large numbers as possible, simply to increase the supply of clinical material and to advance personal interests.

There is certainly some degree of truth in this view; but the whole evil, or even the larger part of it, does not originate from this source. We have been interested observers of the working of both public and private charities for more than forty years; and have never known a place opened for the dispensation of relief to the poor, whether sick or well, that was not besieged by many who were fully able to take care of themselves, or to pay for whatever services they needed.

In the dispensation of ordinary supplies of food and clothing much imposition can be guarded against, by a system of actual visiting and inspection before the applications are answered. But this system cannot be properly and successfully applied, except in a very limited number of cases to applicants for medical advice or medicines.

Is there, then, no way by which the really poor, when sick, can receive prompt and proper attendance and medicines, without opening a door of charity through which thousands will pass who could and should pay for all they need? Is there no way to secure help for the needy and at the same time avoid robbing the profession, the benevolent, and the tax-paying public? So far as attention to the sick is concerned, we think there is. The code of ethics, governing the medical profession, not only permits but enjoins a liberal and faithful attention to the poor when sick, on the part of every member of that profession. And there are in every place, at least in every populous town, physicians enough to supply any sick person in it with all the attention they need. Among them are always a fair proportion of the younger class, well educated, competent, and anxious to accept any call to the sick that will help to fill up their leisure time, add to their practical experience, and further their professional introduction to the community. For this class of practitioners the sick poor are the natural stepping-stones over which they must pass to the paying part of practice. Faithful and skillful attention to the sick poor in their own homes, on the part of young physicians, constitutes the best possible letter of introduction to the rich. But it is not the younger class of physicians only, who promptly and cheerfully render gratuitous service to the sick poor.

During a long professional life we have very rarely known a physician or surgeon of ripe age, full practice and high reputation, to refuse attendance upon the most abject poor when asked, merely because they were poor. Such are compelled sometimes to refuse attendance because previous engagements do not allow a moment of time in which to do it. But even in all such cases they are ready to direct the sufferer to some less occupied professional friend who will attend. Indeed, if every dispensary staff and other organization for furnishing free attention to the sick poor outside of the regular hospitals, were dismissed from duty to-morrow, we do not think there is, or would be, a truly needy sick person in the city who could not get all necessary professional attention by simply asking for it. If these opinions are correct (and of their correctness we have no shadow of doubt), what provisions are actually necessary for the sick poor, and how shall such provision be guarded from abuse?

After devoting much attention to this subject, we are convinced that all establishments, whether called dispensaries, infirmaries, or clinics, where the sick are invited to call for medical advice and attendance, are unnecessary and more or less injurious, both to the profession and the community.

Good public hospitals, to accommodate those who have no homes or places where they can be well cared for when sick, whether poor or not, are necessary. And of these we have a good supply in this city. The only provision needed, outside of such hospitals, is for the proper public authorities (here the county commissioners) to establish from one to three drug depots in each division of the city, supply them with a good assortment of pure drugs, and with a good *dispensing drug clerk* (and assistants if he needs them), to dispense the drugs whenever called for, by the presentation of proper prescriptions. But no prescriptions should be accepted or filled at any of these places unless made by physicians legally authorized to practice medicine in this State, and having plainly written on them that they are given for patients to whom the maker of the prescription gives his own services gratuitously. This would place a supply of reliably dispensed medicines within the reach of all patients, who were sufficiently destitute to satisfy the physician that his own services should be rendered without pay, and at the same time place all those legally authorized to practice medicine on the same footing in relation to the sick poor. The drugs being purchased at wholesale prices, and the depots simple, convenient, inexpensive rooms for dispensing the medicines, the expense of maintaining them would be light, and being accessible for the prescriptions of all physicians on the same terms, there would be no trouble in finding free medical service for every really poor patient asking it. And yet all who could pay even moderate fees for the services of a physician would be compelled to do so, instead of crowding the rooms of dispensaries where each establishment is more anxious to make a large show of attendance, than to make proper discrimination between the deserving and undeserving. Perhaps some one is ready to say that such a system as we have proposed, would deprive the medical schools of a very important part of their material for the clinical instruction of their students. It would be a sufficient answer to such an one, to say that no medical school has any legitimate right to exist, unless so connected with a public hospital, that the necessary clinical instruction can be given within its walls. But the plan we propose would not necessarily prevent the medical colleges from having all the outside clinical material that their faculties could profitably use. Probably not one-third of all those now resorting to the several dispensaries in this city are used for any valuable clinical purpose. And most certainly, the faculty of any college located in the midst of a large city, the members of which were possesed of sufficient ability and reputation to justify them in holding such official position, could draw from the poor within the circle of their own practice and that of their intimate professional friends, all the well selected clinical material they could profitably use, outside of that belonging in the hospitals.

We know the foregoing doctrines in regard to taking care of the sick poor, are not in accord with the fashion of these times. We live in a generation that has very nearly reversed the old rule, "When thou doest thine alms let not thy left hand know what thy right hand doeth." The popular method now is to do everything by organizations admitting of official positions, public meetings, and newspaper heraldings.

It is so much easier to give five, ten, or one hundred dollars, once a year and have it duly acknowledged in the church and the newspapers, and let a salaried officer do the distributing, than it is to personally search for the poor and discriminatingly supply their real wants, and add that friendly counsel which is often more needed than anything else. It is not strange, that in medical matters, the same popular tendencies should have so far predominated, that instead of allowing every practitioner to attend to the sick poor properly within his own circle of practice where he would be led to exercise a just discretion founded on personal knowledge of the parties for whom he acts, the funds should be centered in an organized dispensary fully officered, supplied with a formidable staff both for in-door and out-door attendance, and then if the trumpet is not literally sounded, the *hand-bills* are sent out, with a "Ho! all ye that are ailing, come to the dispensary and get healed without money and without price." Is it any wonder that thousands answer the call who are fully able to take care of themselves, and that in consequence, tax-payers are over-burdened, the mass of the profession robbed of the patronage that belongs to its members, and a premium put on idleness and pauperism? We are glad that the medical aspect of this great social problem is beginning to attract the attention that it deserves. D.

---

PROVIDENT DISPENSARIES.—The Medical Institute of Liverpool refuses to give its countenance to provident dispensaries, because they are business institutions and liable to abuses.

